# Predicting functional associations from metabolism using bi-partite network algorithms

**DOI:** 10.1186/1752-0509-4-95

**Published:** 2010-07-14

**Authors:** Balaji Veeramani, Joel S Bader

**Affiliations:** 1 Department of Biomedical Engineering, Johns Hopkins University, Baltimore, MD21218, USA; 2 High-Through put Biology Center, Johns Hopkins School of Medicine, Baltimore, MD21205, USA

## Abstract

**Background:**

Metabolic reconstructions contain detailed information about metabolic enzymes and their reactants and products. These networks can be used to infer functional associations between metabolic enzymes. Many methods are based on the number of metabolites shared by two enzymes, or the shortest path between two enzymes. Metabolite sharing can miss associations between non-consecutive enzymes in a serial pathway, and shortest-path algorithms are sensitive to high-degree metabolites such as water and ATP that create connections between enzymes with little functional similarity.

**Results:**

We present new, fast methods to infer functional associations in metabolic networks. A local method, the degree-corrected Poisson score, is based only on the metabolites shared by two enzymes, but uses the known metabolite degree distribution. A global method, based on graph diffusion kernels, predicts associations between enzymes that do not share metabolites. Both methods are robust to high-degree metabolites. They out-perform previous methods in predicting shared Gene Ontology (GO) annotations and in predicting experimentally observed synthetic lethal genetic interactions. Including cellular compartment information improves GO annotation predictions but degrades synthetic lethal interaction prediction. These new methods perform nearly as well as computationally demanding methods based on flux balance analysis.

**Conclusions:**

We present fast, accurate methods to predict functional associations from metabolic networks. Biological significance is demonstrated by identifying enzymes whose strong metabolic correlations are missed by conventional annotations in GO, most often enzymes involved in transport vs. synthesis of the same metabolite or other enzyme pairs that share a metabolite but are separated by conventional pathway boundaries. More generally, the methods described here may be valuable for analyzing other types of networks with long-tailed degree distributions and high-degree hubs.

## Background

High quality metabolic reconstructions are available for many organisms and provide a rich scaffold for interpreting data from high-throughput biological experiments. The topology of a metabolic network, defined by connections between enzymes and metabolites, can be used to predict genetic interactions, transcriptional correlations and disease co-morbidity [1-3].

Previous studies have used the topology of the metabolic network to predict co-expression of transcripts for yeast metabolic enzymes [4]. This study first removed high-degree metabolites from the bipartite metabolic network, generated an enzyme-only network by connecting enzymes that shared at least 1 remaining metabolite, and calculated the shortest-path distance between all pairs of enzymes. Shorter distances were correlated with stronger co-expression. Similar procedures, also excluding high-degree metabolites from consideration, were used recently in a study linking diseases to metabolic enzymes [3].

Methods that involve calculation of optimal fluxes subject to constraints, such as flux coupling [5], have performed better than local topological metrics based on shared neighbours in predicting transcript co-expression. Flux coupling methods are much more computationally expensive than topological analysis, however. Furthermore, flux coupling methods suffer from the disadvantage that reactions with small flux values (and hence the enzymes involved in those reactions) are typically removed from the network. This is a problem if an enzyme of interest is removed from the network based on low reaction flux.

Our goal is to provide improved topological measures for enzyme functional associations from metabolic networks without the need for expensive calculations of optimal fluxes or sampling over feasible flux space. The motivation of our approach is that methods that count shared metabolites, or methods that generate a p-value for shared metabolites based on a hypergeometric distribution, essentially assume a flat degree distribution for metabolites. High-degree metabolites violate the assumption of a flat degree distribution, and the hypergeometric distribution is inappropriate for calculating p-values for metabolite sharing. Randomization methods based on rewiring, which maintain the observed degree distribution, are robust to high-degree metabolites but unfortunately are computationally expensive.

In this work, we provide a series of scores that are the Bayesian equivalent of the hypergeometric distribution, but adjusted for the known metabolite degree distributions. These scores are fast to calculate, essentially no more expensive than a hypergeometric p-value, and much faster than any methods that require rewiring permutations, flux sampling, or flux optimization. Results from applying these methods to metabolic networks in yeast demonstrate performance better than previous methods based on local connectedness. The results also reveal functional associations that are not captured by conventional metabolic pathway definitions, but which are inherent in the network structure.

## Results

### Overview

Metabolic networks can be represented as bipartite graphs with edges between enzymes and metabolites. An enzyme can use a metabolite in multiple unique reactions involving distinct subsets of other metabolites, and the number of unique reactions defines an integer-valued edge weight.

**Figure 1:**
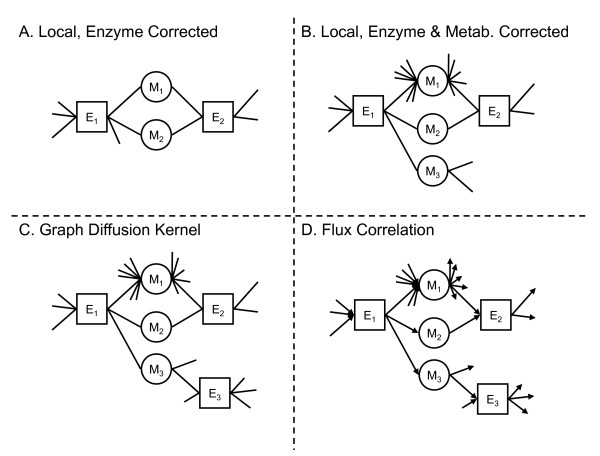
**Methods overview**. A bipartite graph representation of a metabolic network connects enzymes (squares) to metabolites (circles). (A) The number of metabolites shared by two enzymes can be used to rank the functional association of the pair. This raw count can be corrected to account for enzyme degree, using a hypergeometric distribution under a null hypothesis. (B) Hub metabolites, such as M1, can be down- weighted when assessing shared neighbours. One approach is to remove these high-degree metabolites from the network. An alternative threshold-free approach is to incorporate the metabolite degree distribution, as carried out here with a Poisson distribution. (C) One failing of shared neighbour scores is that they are inherently local, providing information only about enzymes that share at least one metabolite. Global methods provide information about all enzyme pairs, no matter how far separated. An improved approach to shortest path between two enzymes is to count paths of all length, with decreasing weight given to longer paths. This method is termed a graph diffusion kernel, with a single parameter used to determine the discount rate for longer paths. (D) Edges in metabolic networks correspond to metabolic fluxes, which have directions and are constrained by maximum capacities and reaction stoichiometry. Functionally related enzymes may have correlated fluxes. The flux correlations can be calculated by repeated sampling, which is computationally intensive but provides a more realistic model for the metabolic network than simpler topological models.

A graphical overview shows how metabolic network information is incorporated to yield increasingly sophisticated models (Figure 1). The models discussed here are all designed to rank pairs of enzymes for functional association. Methods termed "Local" are capable only of producing rankings for enzymes directly connected by at least one metabolite. The raw number of shared metabolites [3,6] and the hypergeometric distribution that corrects the shared count for the enzyme degree (Figure 1A) are both local methods.

In this work, we introduce a more sophisticated local method that also corrects for metabolite degree, discounting the contribution of highly connected metabolites like water, protons, and ATP (Figure 1B). Our new local methods are motivated by Bayesian model selection using the log-likelihood ratio of a null model (random connectivity between enzymes and metabolites) to an alternative model. The number of shared metabolites is modelled as a Poisson distribution for both the null and alternative models. For the alternative model, the Poisson parameter is the maximum likelihood estimate for the observed network, which is the observed number of shared metabolites. For the null model, the Poisson parameter is estimated from a random network model (virtually identical to the leading contribution to the hypergeometric distribution). We present results for an improved Poisson model that uses knowledge of the observed metabolite degree distribution.

Methods termed "Global" are capable of generating rankings for enzymes that are not directly connected by metabolites by using the full network topology. Examples are shortest paths and the more robust graph diffusion kernel (GDK) (Figure 1C). GDKs are non-local in that they sample over all paths between two enzymes, rather than just the shortest paths defined by shared metabolites. GDKs have been successfully applied to functional inference in metabolic networks [7]. Recently parity-specific kernels been used to analyze genetic interaction networks [8]. We also used a method based on the Pearson correlation of the weighted metabolite-enzyme edge connectivity structure between two enzymes.

Yet more elaborate flux balance analysis methods sample flux states that are feasible under steady-state constraints. Flux correlations can then be used to rank enzyme pairs for functional associations (Figure 1D). Other flux balance methods have generated functional associations by predicting synergistic or buffering epistatic interactions for deleting pairs of enzymes from the network.

### Performance of local methods

Performance is assessed primarily by the ability to predict synthetic lethal genetic interactions between metabolic enzymes, and secondarily by the ability to identify classes of enzymes with similar Gene Ontology (GO) annotations. The synthetic lethal interactions provide a direct link to testable experiments. The database annotations are not necessarily testable, but instead show whether inference from computational models is consistent with known biology.

We generated rank ordered lists of enzyme pairs based on the local methods. Performance was assessed from the receiver operating characteristic (ROC) curve using the area under the curve (AUC), and from the precision-recall (PR) curve using the maximum F-score, the harmonic mean of precision and recall. Known positives were taken from experimentally reported synthetic lethal/growth defect interactions recorded in the BioGRID database. There were 170 growth defect/lethal interactions in which both genes involved were part of the metabolic network model. Known negatives were defined as gene pairs where each gene has at least one synthetic lethal interaction, and one of the two has at least 5 synthetic lethal interactions as a query in a high-throughput screen, to exclude pairs that might not have been tested experimentally.

**Figure 2:**
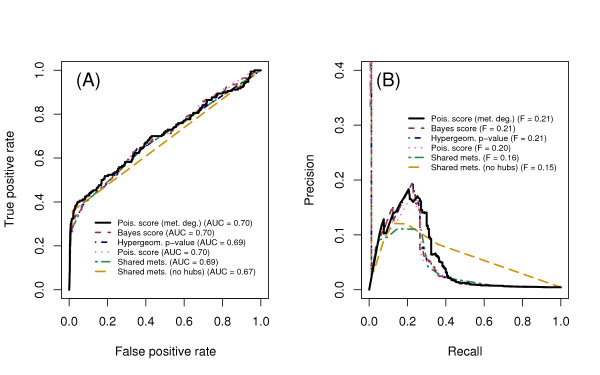
**Performance comparison, local methods**. Methods are listed, from bottom to top, roughly in order of improving performance in predicting synthetic lethal genetic interactions. Shared metabolites indicates raw counts of shared metabolites, and "no hubs" indicates that the most highly connected metabolites were removed before calculating sharing. The Poisson score, hypergeometric p-value, and Bayes score are based on the number of shared metabolites, but do not use the full metabolite degree distribution. The method "Poisson score (met. deg.)" uses the metabolite degree distribution for an additional correction. Performance is assessed using synthetic lethal gene pairs as known positives, and non-synthetic lethal pairs as known negatives. (A) Receiver operator characteristic (ROC) curve. (B) Precision-recall (PR) curve.

**Figure 3:**
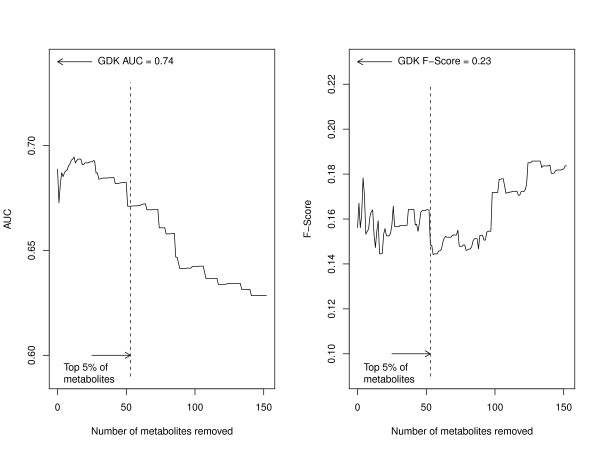
**Performance of raw neighbour count with varying threshold for removing high-degree metabolites**. The degree of each metabolite (the number of reactions it participates in as reactant or product) was calculated, and metabolites were progressively removed. After each removal, the performance of the raw neighbour count for predicting synthetic lethal interactions was assessed by the area under the receiver operating characteristic curve (AUC, panel A) and by the F-Score, defined the maximum harmonic mean of precision and recall along the precision-recall curve (F-Score, panel B). The results in the main text used a nominal threshold of the top 5% of all metabolites, corresponding to 53 metabolites with degree ≥13 of the entire set of 1061 metabolites. The results using a graph diffusion kernel (GDK) are shown to be superior to the raw neighbour regardless of the threshold used.

Performance metrics for the local methods in predicting synthetic lethal genetic interactions are the AUC and F-score (Figure 2). Raw counts of shared metabolites perform the worst. Thresholding the network to remove high-degree metabolites introduces changes in the performance only slightly, and results remain inferior to global methods described below (Figure 3, see [Additional file 1] for details of metabolites removed). The methods that account for the degree of an enzyme, but assume a flat metabolite degree distribution, improve on the raw counts. The new method, which accounts for the metabolite degree as well, performs at least as well as previous methods, and the precision degrades the most slowly of all methods at higher recall.

### Performance of global topology methods

**Figure 4:**
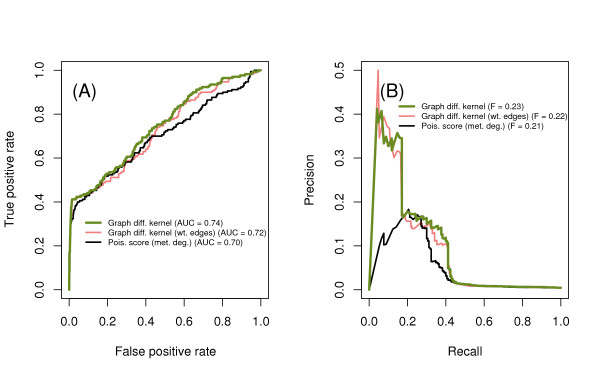
**Performance comparison, local vs. global methods**. The best local method, the Poisson score incorporating metabolite degree, is compared with global methods based on graph diffusion with and without edge weights accounting for multiple reactions for each enzyme. The global methods perform better, and weighted and unweighted diffusion kernels have equivalent performance. (A) ROC curve. (B) PR curve.

We then compared the performance of the best local method (Poisson score using metabolite degree distribution) with methods that use global topology and incorporate local edge weights (Figure 4). Global topology is incorporated using a graph diffusion kernel that sums paths of all lengths between enzyme pairs, rather than just the number of shortest paths involving shared metabolites. Local weights for enzyme-metabolite edges are taken from the integer number of unique reactions involving each enzyme-metabolite pair; these weighted edges were then used to construct a graph diffusion kernel.

Overall, incorporating global information through the graph diffusion kernel improves the performance in identifying synthetic lethal pairs. Adding edge weights to the graph diffusion kernel does not appear to improve performance. Further comparisons with the graph diffusion kernel use the unweighted model only, as it is simpler.

### Global topology with metabolic constraints

We next considered possible improvements that use the knowledge that the network edges represent a flux balance model for metabolism. While others have investigated models that investigate the robustness of metabolism to pairwise gene deletions [9], correlations of fluxes through enzymes provide improved predictions of genetic interactions [2]. We therefore used the flux sampling approach to calculate enzyme correlations, whose absolute values were used to rank enzyme pairs. The flux sampling excludes reactions with negligible flux, which reduces the model to 477 metabolites, 582 reactions and 469 enzymes and reduces the known positive pairs to 69 genetic interactions.

**Figure 5:**
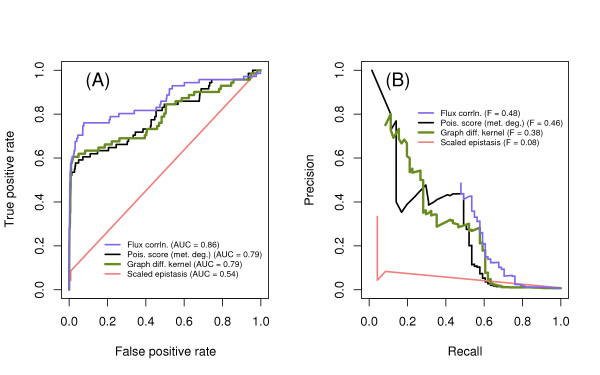
**Performance comparison, incorporating metabolic constraints**. The best local and global methods are compared with methods that use the knowledge that the network models steady-state metabolism. These methods predict synthetic lethal interactions based on decreases to a fitness function ("Scaled epistasis", ref. [10]) and based on correlations of steady-state fluxes sampled over feasible space ("Flux corrln.", ref. [2]). (A) ROC curve. (B) PR curve.

Comparison of the flux sampling method to the best local and global topology methods considers only the enzymes present in the reduced model (Figure 5). The flux sampling method has the best precision in the high-recall region. Enzyme pairs that are used in exactly the same reactions, or which are coupled in an obligate serial pathway, have a flux correlation of 1. These pairs are all ranked identically by the flux sampling method, but may be ranked in a definite order by the local and global topology methods. In the low-recall region, consequently, the local and global methods appear to provide improved precision over the flux sampling approach.

The epistatic estimates, obtained from previous work [9], do not perform as well in predicting synthetic lethality.

### Compartmentalized metabolism

Reactions in the metabolic network are localized to specific compartments (cytoplasm, mitochondria, extracellular, peroxisome, nucleus, Golgi apparatus, endoplasmic reticulum and vacuole). Of the total 1266 metabolic reactions in the model, 47% are entirely contained in the cytosol, roughly 10% are either mitochondrial or extracellular, and 24% couple metabolites from different compartments (Table 1). If one metabolite appears in two different compartments, it is represented as two unique metabolites; enzymes in different compartments that process this metabolite are not scored as sharing it, reducing the ability to detect functional associations for enzymes in different compartments. On the other hand, functional association as measured by synthetic lethality is roughly 3× higher for enzyme pairs that share at least one compartment (Table 2, two-sided p-value = 3 × 10^-6^), and removing compartment information neglects this information.

**Table 1:** Compartment distribution of reactions.

Compartment	Number of reactions
Cytosol	599

Mitochondria	154

Extracellular	122

Peroxisome	58

Nucleus	13

Golgi	6

Endoplasmic reticulum	4

Vacuole	2

Associated with 2 or more compartments	308

Reactions which have metabolites localized to more than one compartment are counted only once and contribute to the total in "Associated with 2 or more compartments".

**Table 2:** Synthetic lethality and shared compartments.

	SL	Non-SL
Share	149	199,644

Do not share	21	81,061

The category "Share" comprises enzyme pairs that have at least one shared compartment based on metabolite localizations (but do not necessarily share a metabolite). The category "Do not share" contains all other pairs.

To test these competing possibilities, we applied the local and global methods to a network in which compartment information was removed. The yeast metabolic network we used in this study specifies compartments for enzymes and metabolites [10]. We reasoned that identical reactions occurring in different compartments can functionally compensate for each other due to diffusion or transport of metabolites across compartment boundaries. We therefore generated a simplified network that ignores the cellular compartments of the metabolites. Removing the compartments reduced the number of metabolites from 1061 to 646.

**Figure 6:**
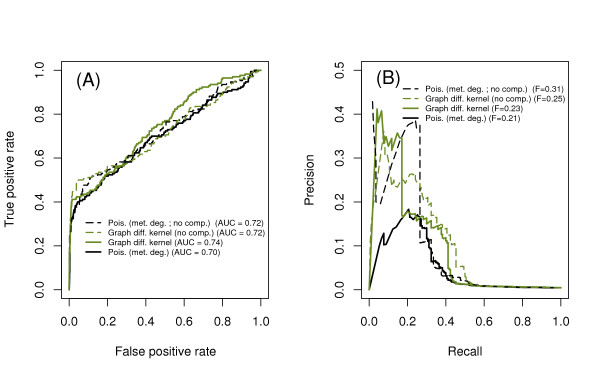
**Performance comparison, compartments**. Local and global methods are tested for the original metabolic network, and for a reduced network in which metabolite compartment information has been removed. (A) ROC curve. (B) PR curve.

The general result for both the local method (Poisson score incorporating metabolite degree) and the global method (graph diffusion kernel with an unweighted network) is that removing compartments improves the precision at high recall at the expense of worse precision at low recall (Figure 6).

### Assessment based on database annotations

We used published methods to assess the ability of different ranking methods to identify enzyme pairs with similar database annotations [11]. This assessment tests the significance of the hypothesis that the average coupling score between all pairs of genes associated with a GO term is higher than between pairs of genes associated with different GO terms. All 835 GO terms mapping to at least 5 and less than 100 enzymes in the network were considered, comprising 538 biological process (BP), 209 molecular function (MF), and 88 cellular compartment (CC). If the null is rejected by the Benjamini-Hochberg procedure at a starting p-value of 0.05/(number of GO terms tested) [12], then the GO term is termed consistent. The fraction of consistent GO terms was computed by this procedure for each method. The assessment was performed for the complete compartment-based network, the reduced compartment-based network with enzymes with negligible fluxes removed, and the network with compartments removed. The category size of 5-100 matches the original publication. Assessment with category sizes of 2-100 and 2-200 yield similar results, but with more categories overall [Additional file 2].

Of all the methods, the graph diffusion kernel clearly performs best overall in producing consistent GO annotations (Table 3). The performance of the methods generally tracks the assessment based on synthetic lethal interaction prediction. Of the local methods, the raw count of shared metabolites performs worst; better are the improved methods that account for enzyme degree; and best of all is the new method that accounts for metabolite degree as well. The graph diffusion method also out-performs the flux sampling method.

**Table 3:** Performance summary statistic (AUC and F-score) and percentage consistent GO terms.

					Percentage of consitent GO terms							
					
Scores	Performance on 170 SL pairs (full network)		Performance on 69 SL pairs(reduced network)		Genes in full network, 283 GO terms (152-BP; 85-MF; 46-CC)				Genes in reduced network, 203 GO terms (110-BP; 60-MF; 33-CC)			
	
	AUC (ROC)	F-Score (PR)	AUC (ROC)	F-Score (PR)	Total	BP	MF	CC	Total	BP	MF	CC
**Compartments**												

Shared Mets	0.69	0.16	0.75	0.26	61	52	69	78	50	41	62	61

Shared Mets (low degree)	0.67	0.15	0.76	0.27	65	64	61	74	55	58	45	61

Hypergeometric p-value	0.69	0.21	0.76	0.43	72	64	79	85	63	55	75	67

Bayes Score	0.70	0.21	0.76	0.43	72	64	79	83	63	55	75	67

Poisson Score	0.70	0.20	0.76	0.40	72	64	79	85	60	52	73	64

Poisson Score (met. degree)	0.70	0.21	0.79	0.47	81	73	94	83	74	65	90	73

Graph diffusion kernel score	**0.74**	0.23	0.79	0.39	**94**	90	**99**	**98**	**92**	**87**	97	**100**

Graph diffusion kernel score (weighted edges)	0.72	0.22	0.79	0.39	93	88	**99**	**98**	**92**	**87**	97	**100**

Flux Correlation	-	-	**0.87**	**0.49**	-	-	-	-	64	66	53	73

Poisson Score (met. associations, deg.)	0.70	0.21	0.79	0.47	81	73	93	83	74	65	92	70

Scaled epistasis	-	-	0.54	0.08	-	-	-	-	-	-	-	-

**No Compartments**												

Shared mets.	0.68	0.09	0.73	0.22	55	47	69	54	49	38	67	52

Shared mets. (low deg. mets.)	0.66	0.18	0.73	0.26	71	72	76	54	64	67	62	58

Hypergeometric P-value	0.69	0.13	0.76	0.39	66	59	78	65	57	45	73	67

Bayes Score	0.69	0.13	0.76	0.39	66	59	78	65	57	45	73	67

Poisson Score	0.69	0.12	0.75	0.33	64	57	76	65	56	44	73	64

Poisson Score (met. degree)	0.72	**0.31**	0.80	**0.49**	78	72	93	72	75	65	92	76

Graph diffusion kernel score	0.72	0.26	0.79	0.38	**94**	**91**	**99**	93	91	85	**98**	94

Graph diffusion kernel score (weighted edges)	0.70	0.25	0.79	0.4	93	90	99	93	89	86	93	88

Poisson score(met. associations, deg.)	0.72	0.30	0.79	0.48	80	74	93	72	75	65	90	79

The best performance in each category is highlighted in bold.

We investigated whether the models with or without compartments perform better using a binomial test under the null hypothesis that each of the two models has equal probability of performing better for a particular method. Tests were performed separately for AUC/ROC, PR/F-score, and the three GO categories, and separately for the full and reduced network. In all cases except GO/Cellular Compartment, two-tailed tests showed no significant deviations from the null hypothesis at p = 0.05. The test for Cellular Compartment is significant (p = 0.0396), with improved consistency for models that include compartment information.

Flux correlation performs best in the Biological Process category. A surprising result here is that overall flux correlation performs worse than GDK and Poisson (metabolite degree, with or without metabolite associations) with only 70% consistent GO terms. It is possible the flux correlation method could be improved with longer runs that reduce the statistical noise in the correlations, although the flux calculations already require over 100× more computer time than any of the other methods (see Computational cost below).

**Figure 7:**
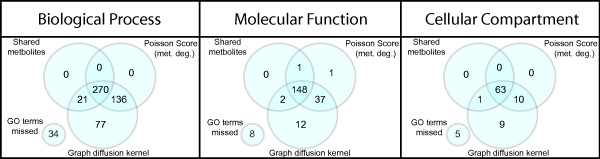
**Consistency of Gene Ontology annotations**. Venn diagrams indicate the number of consistent Gene Ontology (GO) terms identified for Biological Process, Molecular Function, and Cellular Compartment annotations. GO terms that are inconsistent in all three methods are shown as 'GO terms missed'.

The different methods generate a similar set of consistent GO term, and terms missed by the graph diffusion kernel are almost always missed by the other methods as well (Figure 7). For cellular compartment, most annotation terms (72%) are consistent by all three methods. In the case of biological process and molecular function only 50% and 71% of all the annotations are captured by all the three methods.

## Discussion

### Performance of local, global, and flux-based methods

We have investigated the performance of three classes of methods for predicting functional associations in metabolic networks: (i) local methods, based primarily on the metabolites shared by two metabolic enzymes; (ii) global methods, based on the probability that a random walk started at one enzyme will visit a second enzyme; (iii) flux-based methods that use flux balance to identify enzymes with correlated fluxes. The local and global methods are fast and generally applicable to other types of networks, whereas the flux-based methods are computationally expensive and dedicated to metabolism (or other networks that have similar conservation-of-mass constraints). In terms of performance in predicting functional associations, however, the dedicated flux-based methods have typically been superior. Developing fast methods with performance similar to expensive flux-based methods has been a challenge.

Previous local and global methods have had difficulties with high-degree metabolites. For local methods, metabolites such as water and ATP are often shared by enzymes with very different functions. For global methods, these metabolites introduce many short paths through the network. Often, high-degree metabolites are removed from a network prior to analysis. This approach is undesirable because it introduces an ad hoc tuning parameter, which can lead to over-fitting, and it excludes potentially interesting metabolites from the analysis.

The hypothesis motivating this work is that the difficulties from high-degree metabolites arise from an implicit assumption of a narrow metabolite degree distribution, as opposed to the known long-tailed degree distribution. The hypergeometric distribution for shared metabolites corrects for enzyme degree, but not for metabolite degree. Consequently a high-degree metabolite such as water is given the same weight as a low-degree metabolite when counting shared metabolites. Intuitively, high-degree metabolites should be down-weighted. Our improved local method uses the known enzyme and metabolite degrees to generate a degree-corrected score with excellent performance.

The global method we examine, a graph diffusion kernel on the bipartite enzyme-metabolite network, also includes a degree normalization that down-weights the contribution of high-degree metabolites (and high-degree enzymes). This method is somewhat more expensive than the local methods, requiring a full matrix inverse rather than sparse matrix multiplication.

The graph diffusion kernel explores the topology of the metabolic network using random walks that visit metabolites and enzymes. Enzyme-metabolite edges are treated as undirected, permitting random walkers to traverse both directions even for a unidirectional reaction. There are no constraints on the flux of random walkers through any enzyme, and the stoichiometry of a metabolite as a reactant or product is ignored.

Flux-balance methods go beyond graph diffusion by adding constraints specific to metabolic networks. Enzyme fluxes are coupled by mass balance and reaction stoichiometry, and correlations between enzyme fluxes can propagate through the network. These additional constraints capture more of the biological reality of metabolism than either shared metabolites or graph diffusion. Predictive performance is also better, presumably because of the biological constraints. A curious point is that flux sampling, with a uniform sample over the feasible space, performs better than calculations of epistatic effects based on reductions to an optimized fitness objective function. This may indicate errors in the assumed objective function for cellular fitness. The main drawback of flux-balance methods is the high computational cost.

In summary, graph diffusion methods have become a method of choice for analyzing many types of networks. While the degree-corrected local methods provide a substantial improvement over previous local methods, the graph diffusion kernel using the entire network topology performs somewhat better. Dedicated flux-sampling methods are slightly better for predicting genetic interactions, but take over 100× longer to calculate and are much more difficult to implement.

### Disagreement between network-based predictions and database annotations

**Figure 8:**
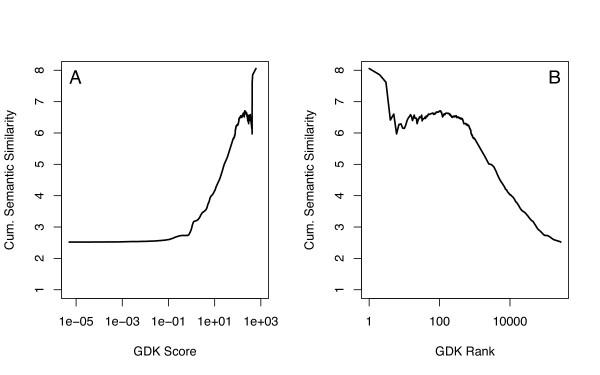
**Cumulative semantic similarity**. The graph diffusion kernel was used to calculate scores for gene pairs (compartment model, diffusion parameter *γ *= 1). For each gene pair, the semantic similarity was also calculated for Biological Process, Cellular Component, and Molecular Function, and the largest of the three values was retained. The cumulative average of this maximum value was then calculated for GDK thresholds of decreasing stringency. (A) The threshold is shown as the GDK score, from least stringent (score = 5 × 10^-6^, the smallest GDK score) to most stringent (617.2, the largest GDK score). (B) The threshold is shown as the rank order, from most stringent (rank = 1) to least stringent (rank = 279,000, the total number of pairs).

As the graph diffusion kernel (GDK) method provides a good trade-off between performance (assessed by genetic interaction prediction) and computational efficiency, it is likely to be a method of choice. The average semantic similarity for gene pairs highly ranked by the GDK provides an additional assessment of performance (Figure 8). GDK scores of 10 and more have an average semantic similarity of 4 or greater, corresponding to no more than 116 genes annotated to the parent category. Thus, on average, a high GDK score indicates a similar database annotation.

**Figure 9:**
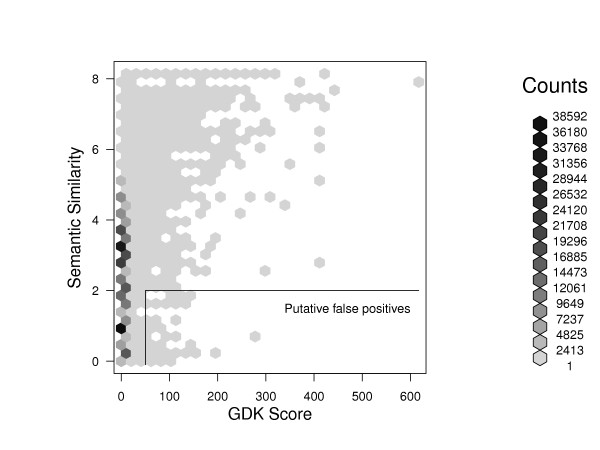
**Putative false positive functional associations**. A density plot of gene pairs binned by semantic similarity and graph diffusion kernel (GDK) score indicates putative false positive functional associations. The semantic similarity for each gene pair was calculated as the maximum of the values for Biological Process, Cellular Component, and Molecular Function. The average of this value over all gene pairs is 2.52, and gene pairs with semantic similarity below this value have essentially no semantic similarity. Gene pairs with GDK scores of 50 and above have an average semantic similarity of 5.6, corresponding to 23 genes annotated to the parent category. Putative false positives were defined for exploratory purposes as having GDK scores of 50 and above but semantic similarity of 2 or below, corresponding to 850 genes annotated to the parent category. The putative false-positive region is indicated and contains 101 gene pairs.

These average results, however, do not always hold for individual gene pairs. A cross-tabulation of semantic similarity vs. GDK score demonstrates that many gene pairs with high GDK scores nevertheless have essentially no semantic similarity (Figure 9). These GDK predictions of functional association would essentially be scored as false-positive predictions based on the lack of similarity in database annotations. Because of the overall good performance of the GDK method, we systematically investigated the most extreme cases in an attempt to suggest a reason for the disagreement.

We therefore selected examples with high GDK scores and low semantic similarity. A GDK threshold of score ≥50 focused attention on the top-ranked GDK pairs, where on average the semantic similarity corresponds to only 23 genes annotated to the parent category of a pair. We then set a threshold of semantic similarity ≤2. This value is substantially below the semantic similarity of gene pairs selected at random, and was chosen to yield a number of example that was feasible for case-by-case analysis, a data set of 101 pairs denoted putative false positives [Additional file 3].

Manual inspection suggests that these cases can be classified into 4 main categories: unannotated, transport-synthesis, pathway-boundary, and secondary activity (Table 4). The unannotated category comprises 10% of the cases and indicates that a gene lacks database annotations for Biological Process, Cellular Component, and Metabolic Function, despite being included in a metabolic reconstruction. The secondary activity category, with only 3 cases, is a related discrepancy in which the secondary metabolic activity of an enzyme is present in the metabolic reconstruction but not noted in GO.

**Table 4:** Categories of gene pairs with high network association and low semantic similarity.

Category	Number of Gene Pairs (of 101)
Transport-Synthesis	66

Unannotated	12

Pathway boundary, Quinone metabolism	7


Pathway boundary, Glycolysis	4

Secondary activity	3

Pathway boundary, N-acetylation	2

Pathway boundary, Purine metabolism	2

Pathway boundary, TCA	2

Pathway boundary, Fumarate metabolism	1

Pathway boundary, Glycoprotein synthesis	1

Pathway boundary, Redox	1

The transport-synthesis category is the largest, with about 65% of the cases. In these examples, the GDK predicts an association between enzymes responsible for synthesis and transport (usually extracellular to intracellular) of the same metabolite. Thus, both enzymes are fulfilling the same role of increasing the intracellular concentration of a metabolite. The structure of the GO annotations does not reflect this close functional association, however. Examples include transport of choline/ethanolime (HNM1-CKI1), allantoate (DAL5-DAL1), sterols (AUS1-ERG27), and uridine (FUI1-URK1).

The final category, pathway-boundary, arises when the boundary between two well-accepted pathways cuts through a metabolite. Enzymes that connect to this metabolite are then annotated to very different pathways, despite a close network-level association. These cases are responsible for 20% of the total. Examples include associations between enzymes in the TCA cycle and those using TCA metabolites for amino acid synthesis, enzymes with different roles in glycoprotein synthesis, and enzymes responsible for quinone metabolism [Additional file 3].

## Conclusions

In analyzing large networks, it has become common to delete high-degree vertices. This practice is questionable. It depends on an arbitrary high-degree cutoff, usually without any clear break in a vertex degree distribution. It can remove vertices that are of interest, and it can introduce unknown biases into the analysis.

Here we have introduced methods that are readily applied to networks with high-degree hubs. Local methods use known degree distributions to correct for high-degree enzymes and metabolites, and global methods use graph diffusion kernels to rank the association between pairs of enzymes. We show that these methods outperform previous methods that eliminate high-degree vertices from the networks. The context is cellular metabolism, where high-degree metabolites like water and ATP are shared by many enzymes. Our methods are able to infer functional associations between enzymes, without being misled by sharing of these high-degree metabolites.

In several cases, enzymes predicted by network analysis to have high functional association have very little similarity in database annotations. Some of these cases are due to a discrepancy between the metabolic reconstruction, which records a reaction for an enzyme, and the annotation database, which lacks information or omits a secondary activity. Two additional patterns were observed, however, which relate to the structure of Gene Ontology hierarchies. First, enzymes that are responsible for synthesis and transport of the same metabolite often have little annotation similarity. Second, conventional pathway definitions may place two enzymes with strong network-level associations on opposite sides of a pathway boundary.

The methods developed here should be applicable in general to other bipartite networks, particularly those with high-degree hubs.

## Methods

### Yeast Metabolic network reconstruction

The Yeast metabolic network used in our study was obtained from the database maintained by systems biology group, University of California, San Diego [10,13]. The file "Sc_-_iND750_-_GlcMM.xml" corresponding to the minimal media condition was obtained from http://gcrg.ucsd.edu/Downloads/Cobra_Toolbox. This network has 1061 metabolites, 1266 reactions and 750 genes. The stoichiometry matrix *S*(*m*, *r*) provides the number of metabolites *m *consumed or produced in reaction *r*. The reaction-gene association matrix *E*(*r*, *e*) in the metabolic network indicates whether reaction *r *can be catalyzed by enzyme *e*.

### Coupling measures based on metabolic bipartite network

A bipartite network has two disjoint sets of vertices with edges only between vertices of different sets. In the case of the metabolic network, we consider enzymes *e *and the metabolites *m *as disjoint vertices in a bipartite graph. We use various metabolic coupling measures between two enzymes in this graph to predict synthetic lethal genetic interactions. Towards this goal, we use both methods from literature (based on shared metabolites [6], shared metabolites after removing high degree metabolites [3,4]) and other methods proposed here.

### Shared metabolite count (with and without hubs)

The coupling between metabolites can be calculated using the stoichiometry matrix as $\hat{S}{\hat{S}}^{T}$[6]. The elements of $\hat{S}$, denote the participation of a metabolite *i *in a reaction *j *with a value 1 and 0 otherwise ($\hat{S}$ is binary version of the stoichometry matrix, S). This idea extended to the coupling of genes based on the bipartite metabolic network could be represented as $\overset{\wedge }{M}{\overset{\wedge }{M}}^{T}$, where *M^T ^*= $\hat{S}$*E*, and $\hat{M}$ is the binary version of the matrix *M*. The element *C*_*ij *_of the matrix *C *now represents number of metabolites shared between enzyme *i *and enzyme *j*.

The metabolite degree is defined as the number of reactions in which a metabolite participates. In previous work, high-degree metabolites have been excluded from metabolite sharing (equivalent to ignoring the rows corresponding to metabolite hubs in the stoichiometry matrix) [3]. In our calculations of shared metabolites, the top 5% of metabolites were excluded (53 metabolites participating in 13 or more reactions).

### Bayesian score

We used our previous method based on 2 × 2 contingency table for calculating the metabolites shared between two enzymes [2]. Briefly, this measure is obtained as a log likelihood ratio of alternative to null hypothesis. Under the null, the probability of connection of a metabolite to both enzymes is product of the individual probabilities of connections. Let *n, n*_1_, *n*_2 _*and n*_12 _represent the total number of metabolites in the network, the number connected to enzyme 1, the number connected to enzyme 2, and the number connected to both. The score is then

(1) Bayesian Score=log[(n+1)·C(n,n2)(n+2)·(n+3)·C(n1,n12)]−log[C(n−n1,n2−n12)].

The combinatorial factor *C(n*, *k) is n*!*/k*!(*n - k*)!. This score increases when *n*_12 _is either larger or smaller than the value *n*_1_*n*_2_/*n *expected under the null hypothesis (analogous to a two-sided test). For *n*_12 _smaller than the null expectation, we used score(*n*_12_) - |score(*n*_12_) - score(*n*_1_*n*_2 _/ *n*)| to restrict attention to enrichment.

### Hypergeometric p-value score

The score based on the hypergeometric p-value for characterizing the metabolic coupling between the enzymes 1 and 2 is

(2) Hypergeometric p-value score=−log[∑k=n12min(n1,n2)C(n1,k)·C(n−n1,n2−k)C(n,n2)].

### Poisson score

This score is also obtained as the log-likelihood ratio of an alternative to null model for the observed number of shared partners. Both the alternative and the null employ a Poisson distribution with a single parameter *λ*. For the alternative, *λ*_alt _= *n*_12_, the observed count; for the null, *λ*_null _= *n*_1_*n*_2_/*n*_tot_. The total number of metabolite-enzyme edges is *n*_tot_; *n*_1 _and *n*_2 _are the numbers of metabolites connected to each enzyme; and *n*_12 _is the intersection of the metabolites in *n*_1 _and *n*_2_. The Poisson score is

(3) Poisson Score=n12·log[n12n1·n2/ntot]−|n12−n1·n2ntot|.

The absolute value for the second term in Eq. 3 ensures that large scores come from enrichment rather than depletion of shared metabolites.

### Poisson score with metabolite degree

The null model in the Poisson score was further improved by considering a different value for *λ*_null _using the degree distribution of the metabolites connected to the enzymes. Let *k*_*i*1_, *k*_*i*2_,..., $k_{in_{i}}$ be the degree of the metabolites *m*_*i*1_, *m*_*i*2_,..., *m*_*in *_connected to enzyme *i *in the enzyme pair (*i *= 1,2). The probability of the metabolite *m*_11 _to be connected to enzyme 2 is

(4) Pr(m11~ Enzyme 2)=1−[1−n2ntot]k11≈1−exp[−k11·n2ntot].

The average number of metabolites connected to enzyme 1 that are also connected to enzyme 2 is then

(5) $$\lambda_{\text{null}}^{12}=\sum_{i=1}^{n_{1}}\left[1-\exp\left(-\frac{k_{1i}\cdot n_{2}}{n_{\text{tot}}}\right)\right].$$

The values of $\lambda_{\text{null}}^{12}$ and $\lambda_{\text{null}}^{21}$ are in general different due to different degree distribution of the metabolites connected to enzymes 1 and 2. The value of *λ*_null _used in the improved null model is obtained by arithmetic mean of $\lambda_{\text{null}}^{12}$ and $\lambda_{\text{null}}^{21}$. The final expression for the improved Poisson score is

(6) Poisson Score(met. degree)=n12·log[n120.5·λnull12+0.5·λnull21]−|n12−0.5⋅λnull12−0.5⋅λnull21|.

### Poisson score with metabolite associations and degree

A further improved Poisson score can be generated using a more elaborate *λ*_null_, accounting for the full metabolite degree distribution. The probability of *k *connections given the status of the metabolites *m*_1_,..., *m*_i _connected to enzyme *E*, *P*^*E*^(*k*|*m*_1_,..., *m_i_*) is

(7) PE(k|m1,...,mi)=PE(k|m1,⋯,mi−1)·P(E≁mi|k)+PE(k−1|m1,...,mi−1)·P(E~mi|k−1).

The probability of enzyme connected and not connected to the metabolite *m_i _*are estimated as

(8) P(E~mi|k−1)=1−exp(−ki·(nE−k+1)ntot)P(E≁mi|k)=exp(−ki·(nE−k)ntot).

We take *P^E^*(*k *= -1|*m*_1_,..., *m_i_*) = 0, *P^E^*(*k *= 0|-) = 1 in the calculations and obtain *P^E^*(*k*|*m*_1_,..., *m_i_*) for the enzymes A and B in the pair (and respectively connected to *n*_1 _and *n*_2 _metabolites) to obtain the *λ*_null_,

(9) λnull=0.5⋅∑k=0min(n1,n2)k⋅PA(k|m1,...,mn1)+0.5⋅∑k=0min(n1,n2)k⋅PB(k|m1,...,mn2).

We use *λ*_null _(Eq. 9) to obtain the Poisson score that takes metabolite associations and degree into account,

(10) Poisson Score (met. association, degree)=n12·log[n12λnull]−|n12−λnull|.

Results from this more complicated model are included in Table 1, but not discussed in the text as the method is more complicated yet performs no better than simpler Poisson score methods.

### Graph diffusion kernel

Graph diffusion kernels are solutions to the steady-state density distribution for continuous-time random walk or diffusive process on a graph with sources and sinks [8]. The adjacency matrix for the calculation of the GDK measure (Eq. 11) has a block structure whose dimension is given by the sum of number of metabolites and enzymes in the yeast metabolic network (i.e. 1061+750 = 1811),

(11) $$A=\left[\begin{array}{cc} 0 & {\overset{\wedge }{M}}^{T}\\ \overset{\wedge }{M} & 0 \end{array}\right].$$

The matrix $\hat{M}$ is the binary version of the matrix *M *defined as *M*^*T *^= $\hat{S}$*E *and captures the direct links from metabolites to enzymes. The degrees of the nodes are summarized by the diagonal matrix D (D_*ii *_= ∑_j _A_ij _, with A_ij _the elements of the adjacency matrix, A). The graph diffusion kernel score with normalization for the node degrees is

(12) Graph diffusion kernel score=[(1+γ)·I−D−12AD−12]−1.

The parameter *γ *controls the extent of diffusion, or equivalently the length of the random walks. These lengths are distributed exponentially, with the probability of a *d*-step walk proportional to e-^*γ*d^. The results shown in this work are for a value of *γ *= 1. Results were not sensitive to the value of *γ*, with similar results over a range from 0.5 to 120. The entries in the kernel corresponding to the enzyme-enzyme relationships were then extracted to predict genetic interactions. For readability, GDK scores displayed in the figures are multiplied by 10^4^.

### Graph diffusion kernel with weighted edges

We also considered a version of the graph diffusion kernel with weighted edges. Here we used the full version of the matrix *M*(*M*^*T *^= $\hat{S}$*E) *in the adjacency matrix *A *rather than a binary version as used in the non-weighted case (Eq. 11). The element *(M^T ^)_ij _*of the matrix *M*^*T *^= $\hat{S}$*E *represents the number of times a metabolite *i *is associated with the enzyme *j *through various reactions. Then kernel score with weighted edges is obtained using the same procedure as described above. Results are shown for *γ *= 1 and remained the same for higher *γ *values.

### Scores without compartments

The model obtained from the BIGG database is a fully compartmentalized model with same metabolites localized to different compartments represented separately. Some metabolites may move between compartments freely, others through ion-channels by chemical gradients or through transporters. This may bring the reactions that use the metabolites that diffuse freely in different compartments closer in that they share either the substrates or products. To investigate the effect of this in our analysis, we combined same metabolites localized to different compartments (by adding the rows of the stoichometric matrix). Then we calculated all the metabolic coupling measures (except enzyme flux correlation measure) with the compartment-free metabolic network. There were 646 metabolites in the compartment-free network.

### Enzyme flux correlation

The performance of various scores considered in this work were compared with enzyme flux correlation score used in our previous study [2]. Briefly, the method is based on feasible reaction fluxes obtained under stoichometric and reaction flux constraints at steady state [14]. The set of feasible reaction fluxes were sampled using a Markov random sampling algorithm under *in silico *medium similar to YPD [15]. Then reaction fluxes were transformed to enzyme fluxes. The enzyme flux correlation between two enzymes is then obtained by calculating the Pearson correlation coefficient over the various enzyme flux samples. The details of the calculations are available elsewhere [2]. This entire procedure from random sampling to calculating correlations was repeated 3 times with different random seeds, with no evidence of non-ergodic sampling among the three runs. Final predictions used absolute value of correlation averaged over three runs. A preliminary step before random sampling removes all blocked reactions which carry no flux. The reduced model used for flux sampling had 477 metabolites, 582 reactions and 469 enzymes. The flux sampling procedure was carried out with the COBRA MATLAB toolbox [16]. The absolute value of the flux correlation is used for ranking.

### Scaled epistasis

The scaled epistasis values corresponding to the minimal media were obtained from a previous study [9]. The file "fitness_data_nominal.txt" containing the fitness of insilico single and double gene yeast knockouts were obtained from http://kishony.med.harvard.edu/prism/index.html. For calculating the AUC and F-score from scaled epistasis score, only enzyme pairs with epistasis score were considered. We did not calculate GO consistency scores for this method because it performed poorly for predicting genetic interactions.

### Synthetic lethality data sources

Synthetic lethality data for this study was obtained from the BioGRID database (version 2.0.46) [17]. There were 97 synthetic lethal and 73 synthetic growth defect interactions in the BioGRID database that had both the genes in the yeast metabolic network. There were 39 synthetic lethal and 30 growth deflect interactions in the BioGRID database that had both the genes in the reduced model used in the flux sampling procedure.

### Performance metrics

The ROC curves, AUC, F-score and PR curves were generated in R using the ROCR package [18]. The ROC and PR curves are shown with a downsampling option in the plot set to 5000.

### Gene Ontology assessment

We used a procedure proposed in a previous study for validating the functional gene similarity measures [11]. We used gene ontology (GO) annotation terms from all the three categories biological process, cellular compartment and molecular function. A GO term is termed consistent if the average metabolite coupling score between all pairs of metabolic genes associated with the GO term is greater than genes that do not share a same annotation.

The percent of consistent GO terms were calculated for each bipartite coupling measure. We considered only GO terms associated with 5 though 100 genes. For each GO term, the pairwise coupling score between metabolic genes associated with it are averaged. The statistical significance of this averaged score is assessed by random shuffling of gene GO annotation associations, maintaining both the annotation and gene distribution. We calculated an empirical p-value based on 10,000 iterations for each GO term. These empirical p-values were corrected for multiple testing of many GO terms to control for false discovery rates [12]. The consistency score was obtained by the proportion of GO terms that were significant with a false discovery rate of 0.05. The GO gene associations of yeast corresponding in the file gene_-_association.sgd was obtained from the Saccharomyces genome database, http://downloads.yeastgenome.org/literature_curation/.

### Semantic Similarity

Semantic similarity was calculated as *l*_*n*_*(N*_*T*_*/N*_*P *_*)*, where the total number of genes *N*_*T *_= 6310 for yeast, and the number of genes annotated to the closest parent category of two genes is *N*_*P *_[19,20]. Semantic similarity values were calculated separately for the three main Gene Ontology hierarchies: Biological Process, Cellular Component, and Metabolic Function. These three values were then summarized by the maximum of the three to identify functional associations inferred from network structure that do not match any known annotation similarity.

### Computational cost

The methods proposed in this work are computationally less expensive as compared to the flux correlation based approaches. The computation of flux correlation takes about 15 hours (each sampling run taking about 5 hours). Computing the other scores was performed as a single calculation that required only 9 minutes. The graph diffusion kernel, part of this single calculation, was computed directly as the inverse of the graph Laplacian rather than as a repeated matrix multiplication.

## Authors' contributions

BV and JSB conceived the study and wrote the manuscript. BV performed the work. All authors read and approved the final manuscript

## Supplementary Material

Additional file 1 **Performance of raw neighbor count**. The data from Figure 3 are replotted with the addition of the name, cellular localization (c = cytoplasm, m = mitochondrion, n = nucleus, x = extracellular), and degree of each metabolites at the position it is removed. Click here for file

Additional file 2 **Consistent GO terms and category size**. GO consistency analysis results are provided for category sizes of 2-100 and 2-200, compared with 5-100 in the main text. Click here for file

Additional file 3 **Putative false positives**. The 101 gene pairs with high network association and low semantic similarity are tabulated and the reasons for disagreement are annotated. Click here for file
