# Supplementary material for: Predicting functional associations from metabolism using bi-partite network algorithms
Source: BMC Syst Biol. 2010 Jul 14;4:95. doi: 10.1186/1752-0509-4-95 (PMC2919484; doi:10.1186/1752-0509-4-95)
Supplement: Additional file 2 — Consistent GO terms and category size. GO consistency analysis results are provided for category sizes of 2-100 and 2-200, compared with 5-100 in the main text. [file 1752-0509-4-95-S2.PDF]

Category Size	Ont	SM	PS	GDK	SM-PS	PS-GDK	SM-GDK	All	None	Total
5-100	BP	0	0	99	0	140	26	281	34	580
	CC	0	0	9	0	10	1	72	5	97
	MF	0	2	12	1	39	3	163	7	227
2-100	BP	0	9	153	0	193	21	407	163	946
	CC	0	0	11	0	21	1	121	15	169
	MF	1	11	18	4	73	4	291	82	488
2-200	BP	0	12	178	2	191	26	421	158	988
	CC	0	1	15	0	17	1	129	15	178
	MF	0	17	18	5	71	4	310	81	506

Additional File 2. Sensitivity of Consistent GO Terms to Category Size

Category size: Number of genes for GO categories considered for consistency analysis (endpoints inclusive). The 5-100 range is discussed in the main text and presented as Venn diagrams (Fig. 7). This range used 2000 permutations to generate a null distribution. The 2-100 and 2-200 ranges used 500 permutations to generate a null distribution.

Ont: Ontology, Biological Process (BP), Cellular Compartment (CC), or Molecular Function (MF)

The remaining columns provide counts for regions in the corresponding Venn diagrams for each category size range and ontology.

SM: Found only by Shared Metabolites score
PS: Found only by Poisson score
GDK: Found only by Graph diffusion kernel score

SM-PS: Found by SM and PS but not GDK
PS-GDK: Found by PS and GDK but not SM
SM-GDK: Found by SM and GDK but not PS

All: Found by SM, PS, and GDK

None: Found by no methods

Total: All GO terms for the indicated ontology and range
