# Supplementary material for: Predicting functional associations from metabolism using bi-partite network algorithms
Source: BMC Syst Biol. 2010 Jul 14;4:95. doi: 10.1186/1752-0509-4-95 (PMC2919484; doi:10.1186/1752-0509-4-95)
Supplement: Additional file 1 — Performance of raw neighbor count. The data from Figure 3 are replotted with the addition of the name, cellular localization (c = cytoplasm, m = mitochondrion, n = nucleus, x = extracellular), and degree of each metabolites at the position it is removed. [file 1752-0509-4-95-S1.PDF]

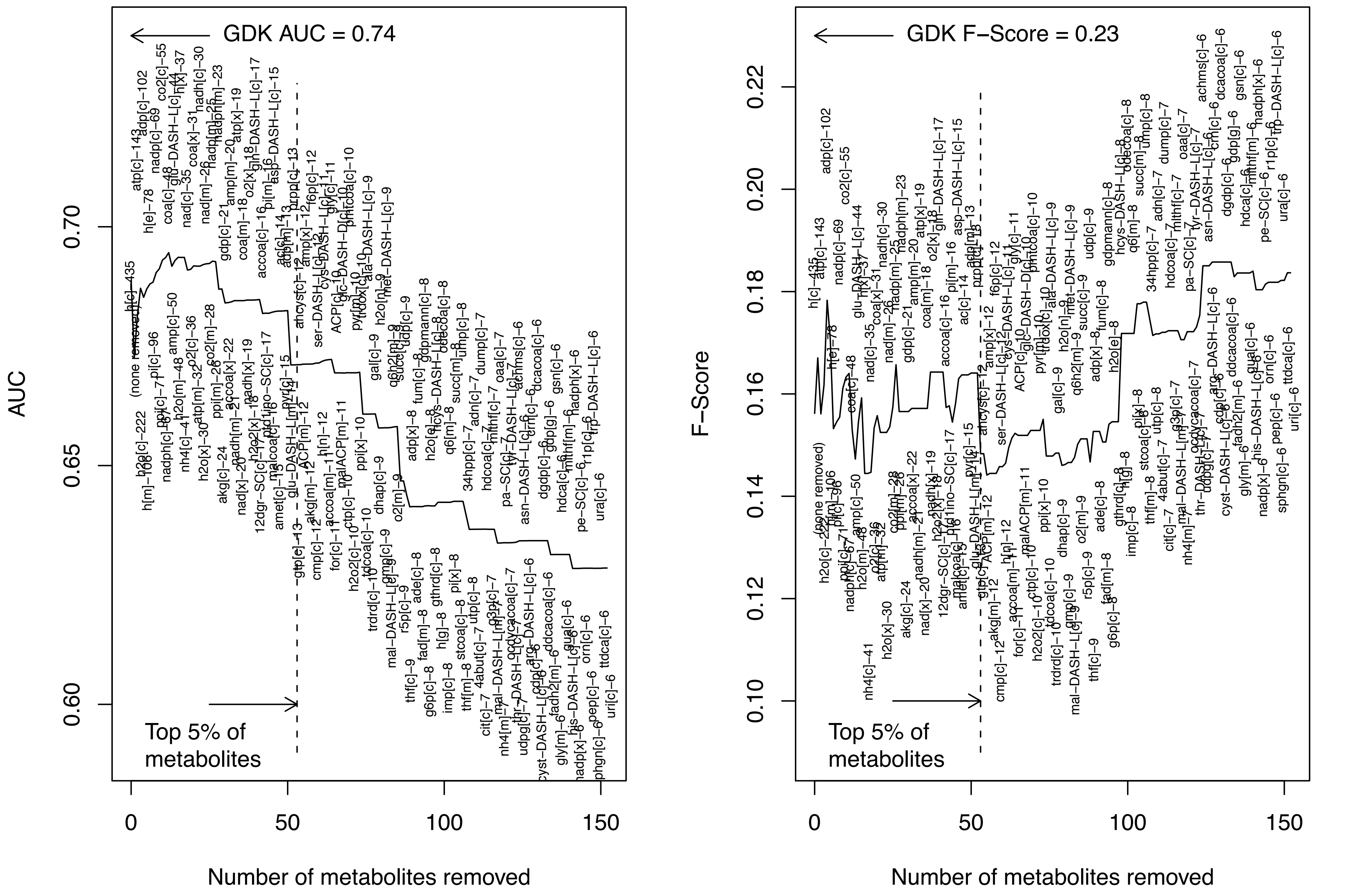
GDK AUC = 0.74
AUC
0.60
0.65
0.70
Top 5% of
metabolites
Number of metabolites removed
0
50
100
150
(none removed)
GDK F−Score = 0.23
F−Score
0.10
0.12
0.14
0.16
0.18
0.20
0.22
Top 5% of
metabolites
Number of metabolites removed
0
50
100
150
